# Predicting Sustained Clinical Response to Rituximab in Moderate to Severe Systemic Manifestations of Primary Sjögren Syndrome

**DOI:** 10.1002/acr2.11466

**Published:** 2022-06-05

**Authors:** Sophanit Pepple, Jack Arnold, Edward M. Vital, Andrew C. Rawstron, Colin T. Pease, Shouvik Dass, Paul Emery, Md Yuzaiful Md Yusof

**Affiliations:** ^1^ Leeds Institute of Rheumatic and Musculoskeletal Medicine University of Leeds Leeds UK; ^2^ NIHR Leeds Biomedical Research Centre Leeds Teaching Hospitals NHS Trust Leeds UK; ^3^ Haematological Malignancy Diagnostic Service Leeds Teaching Hospitals NHS Trust Leeds UK

## Abstract

**Objective:**

To assess outcomes of repeat rituximab cycles and identify predictors of sustained clinical response in systemic manifestations of primary Sjögren syndrome (pSS).

**Methods:**

An observational study was conducted in 40 rituximab‐treated patients with pSS. Clinical response was defined as a 3‐point or more reduction in the European League Against Rheumatism (EULAR) Sjögren Disease Activity Index (ESSDAI) at 6 months from baseline. Peripheral blood B cells were measured using highly sensitive flow cytometry. Predictors of sustained response (within two rituximab cycles) were analyzed using penalized logistic regression.

**Results:**

Thirty‐eight out of 40 patients had moderate to severe systemic disease (ESSDAI >5). Main domains were articular (73%), mucocutaneous (23%), hematological (20%), and nervous system (18%). Twenty‐eight out of 40 (70%) patients were on concomitant immunosuppressants. One hundred sixty‐nine rituximab cycles were administered with a total follow‐up of 165 patient‐years. In cycle 1 (C1), 29/40 (73%) achieved ESSDAI response. Of C1 responders, 23/29 received retreatment on clinical relapse, and 15/23 (65%) responded. Of the 8/23 patients who lost response, these were due to secondary non‐depletion and non‐response (2NDNR; 4/23 [17%] as we previously observed in systemic lupus erythematosus with antirituximab antibodies, inefficacy = 2/23, and other side effects = 2/23). Within two cycles, 13/40 (33%) discontinued therapy. In multivariable analysis, concomitant immunosuppressant (odds ratio 7.16 [95% confidence interval: 1.37–37.35]) and achieving complete B‐cell depletion (9.78 [1.32–72.25]) in C1 increased odds of response to rituximab. At 5 years, 57% of patients continued on rituximab.

**Conclusion:**

Our data suggest that patients with pSS should be co‐prescribed immunosuppressant with rituximab, and treatment should aim to achieve complete depletion. About one in six patients develop 2NDNR in repeat cycles. Humanized or type 2 anti‐CD20 antibodies may improve clinical response in extra‐glandular pSS.

## INTRODUCTION

B cells play a major role in the pathogenesis of primary Sjögren syndrome (pSS) through the production of autoantibodies directed against SS‐A/Ro and/or SS‐B/La, elevated levels of rheumatoid factor, hypergammaglobulinemia, elevated levels of free light chains, and increased risk of non‐Hodgkin lymphoma (1, 2, 3, 4). Thus, B‐cell–targeted therapy presents a logical therapy. Despite some evidence of rituximab effectiveness in relation to the patient‐reported outcomes from open label studies (5, 6, 7, 8) and pilot, double‐blind, placebo‐controlled randomized controlled trials (RCTs) (9, 10), two pivotal phase 3 RCTs (the Tolerance and efficacy of rituximab in primary sjogren syndrome [TEARS], which evaluated a single course of rituximab (11) and the A Trial of anti‐B‐cell Therapy in Patients with Primary Sjögren's Syndrome [TRACTISS], evaluating two courses of therapy (12)) failed to meet their primary endpoints. Therefore, do B‐cell–targeted therapies still have a place for the treatment of pSS?

It is worth noting that the outcome measures used in both RCTs above might not be sufficiently sensitive to changes in the patients’ clinical and biological response to B‐cell–depleting therapy. The primary endpoints for both trials above were assessed using the patient‐reported visual analogue scale measuring fatigue and dryness in the TRACTISS (12), as well as pain and patient's global assessment of disease activity in the TEARS (11). Interestingly, objective measures such as improvement in unstimulated salivary flow rate were greater in the rituximab versus placebo groups in the TRACTISS. Post‐hoc analyses using ultrasound of salivary glands in the TEARS also reported greater improvement in the rituximab versus placebo groups with regard to the salivary gland echostructure (13) and the baseline‐adjusted total ultrasound score in the TRACTISS (14). Moreover, transcriptomic and histological analyses of salivary gland biopsy samples from the TRACTISS reported beneficial effects of rituximab over placebo in reducing the progression of B‐cell–driven inflammatory infiltrate by downregulating genes involved in immune cell recruitment, activation, and organization in ectopic germinal center (15). Although there was no difference observed in the composite index, the European League Against Rheumatism (EULAR) Sjögren Syndrome Disease Activity Index (ESSDAI) between the rituximab and placebo groups (apart from at week 36) in the TRACTISS, the patients recruited in this study had generally low ESSDAI scores at baseline (ie, mean 5.3 ± 4.7 for the rituximab group relative to the maximum score of 123) (12).

In addition to the issue with outcome measures, failure of these trials to meet their primary endpoints also could be attributed to unstratified patient selection of a disease with heterogeneity in clinical features. Some studies reported effectiveness of rituximab in patients with pSS with systemic involvement (8, 16). More data and definitive studies are warranted for this subgroup of patients, perhaps with the greatest unmet need of effective systemic therapies. Recent data have also demonstrated the existence of four immunologically and clinically distinct strata in pSS. Reanalysis of the TRACTISS data based on these four groups showed significantly greater improvement in both the unstimulated and stimulated salivary flow rates in patients assigned to the dryness dominant with fatigue (DDF) group compared with the placebo. These patients had the highest mature B‐cell transcriptomic modular score and would be expected to respond best to a B‐cell–targeted agent (17).

Even if B cells are an appropriate therapeutic target, existing rituximab‐based protocols may not always block this target adequately. We previously showed the association between achieving complete B‐cell depletion and clinical response in rheumatoid arthritis (RA) (18) and systemic lupus erythematosus (SLE) (19, 20) when peripheral B‐cell subsets were enumerated using highly sensitive flow cytometry (HSFC). Because insufficient depth or duration of B‐cell depletion may explain poor clinical responses, more potent next‐generation CD20 therapies may be more effective. Surprisingly, there are no documented B‐cell biomarkers of response in pSS.

In patients with pSS who respond to the initial course of rituximab, there are limited data on the outcomes of subsequent and repeated courses of therapy. In SLE, we and others previously reported a phenomenon called secondary non‐depletion and non‐response (2NDNR), whereby patients who initially responded well to rituximab with B‐cell depletion subsequently experienced a severe infusion reaction longer than 24 hours during the second infusion of a cycle, failed to completely deplete CD20+ B cells and did not clinically respond during repeat cycles. 2NDNR was associated with antirituximab antibodies (20, 21, 22, 23). This phenomenon has also been described in patients with rituximab‐treated anti‐neutrophil cytoplasmic antibody‐associated vasculitis (AAV) (24).

In the present study, our objectives were to assess the outcomes of repeat cycles of rituximab and identify predictors of sustained clinical response with a view to personalized future development of CD20‐depleting therapies in extra‐glandular pSS.

## PATIENTS AND METHODS

### Patients and design

A retrospective observational cohort study was conducted of the first 1000 consecutive rituximab‐treated patients with any rheumatological diagnosis in a single center between December 2004 and May 2021. Inclusion criteria were being an adult (≥18 years old), fulfilling the revised 2002 American‐European Consensus Group classification criteria for pSS (25), and having at least a 6‐month follow‐up post rituximab. Exclusion criteria were having secondary Sjögren syndrome and concurrent anti‐cyclic citrullinated peptide antibody positivity. For cross‐disease comparison, B‐cell subsets of patients with pSS were compared with our previously published cohort of RA (N = 111) (26), SLE (N = 117) (20), and AAV (N = 70) (27, 28).

### Ethical approval

A formal ethical approval was not required because all treatment decisions were made prior to evaluation of data, in accordance with the National Health Service (NHS) Research Ethics Committee guidelines. The use of off‐label rituximab was approved by the Leeds Teaching Hospitals NHS Trust Drug and Therapeutic Committee.

### Treatment

All patients received a first cycle of therapy consisting of 100 mg of methylprednisolone and 1000 mg of rituximab given intravenously on days 1 and 14. Of these, 30 patients received MabThera, whereas 10 were treated with Truxima. Further cycles consisting of the same regimen were repeated on clinical relapse (defined below). Continuation of a stable dose or reduction of concomitant immunosuppressant including oral prednisolone was left to clinicians’ discretion, aiming to stop glucocorticoid if remission was achieved at 6 months.

### Clinical data and outcomes

Disease activity was assessed at baseline and every 6 (+3) months post‐rituximab using the ESSDAI (29). ESSDAI was scored retrospectively before January 2018 and prospectively thereafter in clinic. Data were gathered thoroughly and extensively from various sources including electronic health records, hospital admission records, pathology results server, clinic correspondences, and medical case notes. The cryoglobulin test was not performed in all patients and only requested in those with suspected cryoglobulinemia and abnormality in either rheumatoid factor or complement level. Cryoglobulinemia was considered not present in those without test results data. Clinical response was defined as a 3 or more point reduction from baseline (30). Relapse was defined as new, reappearing, or worsening of persistent disease.

### Routine laboratory measures

Antinuclear antibody was measured by indirect immunofluorescence. Anti‐double‐stranded DNA titers and extract nuclear antigens profile (anti‐Ro (60), ‐Ro (52), ‐La, ‐Sm, ‐Scl‐70, ‐Jo‐1, ‐RNP, ‐Sm/RNP, ‐Ribosomal P, ‐Chromatin) were measured using ImmunoCAP chemiluminescent immunoassay by Thermo Fisher Scientific prior to July 2012 and Bioplex 2200 Immunoassay (after July 2012). Complement levels (C3 and C4; normal range for C3: 0.75–1.65 g/L and for C4: 0.14–0.54 g/L) and immunoglobulin titers (normal range for immunoglobulin M [IgM]: 0.5–2.0 g/L; IgA: 0.8–4.0 g/L; and IgG: 6.0–16.0 g/L) were measured by nephelometry. All immunological tests above were measured at baseline and every 6 months post‐rituximab to comply with the Rituximab Funding requirement for evidence of effectiveness at an accredited NHS laboratory.

### Peripheral blood B cells

Peripheral blood B‐cell subsets (naïve, memory, and plasmablast cells) were enumerated using HSFC as previously described (18) at weeks 0, 2, and 26 without knowledge of clinical status other than time since rituximab. B cells were measured as a part of routine clinical practice in our department at an accredited Leeds Haematological Malignancy Diagnostic Service laboratory. A six‐color flow cytometry protocol (CD3, CD14, CD19, CD27, CD38, and CD45) counting 500,000 events was used. Naïve (CD19 + CD27−), memory (CD19++CD27+), and plasmablast (CD19+/−CD27++CD38++) counts were enumerated using CD45 to identify the total leucocyte population for calculation of absolute B‐cell subset numbers, using CD3 and CD14 to exclude contaminating leucocyte populations. Complete B‐cell depletion was defined as a total B‐cell count of less than 0.0001 × 10^9^ cells/L and repopulation as counts greater than or equal to 0.0001 × 10^9^ cells/L.

### Statistical analyses

Descriptive statistics were summarized using mean with SD or median with interquartile range (IQR) for continuous variables and proportion for categorical variables. The significance of the association between categorical variables was tested by Fisher's exact test (expected value <5) or χ^2^ test (if otherwise), whereas for continuous variables the Mann‐Whitney U test was used. Comparison of B‐cell subsets between four diseases were analyzed using the Kruskal‐Wallis test, followed by multiple non‐parametric testing between groups with Dunn's correction. Multiple imputation by chained equations was used to estimate missing data, and 20 multiple imputation sets were used to provide stability of results. For the prediction of rituximab short‐term response (ie, within the first two cycles), this was analyzed using multivariable penalized logistic regression in order to minimize overfitting of data (31). Penalized logistic regression with multiple imputation were undertaken using forward selection and backward elimination, with *P* < 0.20 associated with the deviance used for inclusion in and exclusion from the model. For the prediction of time to rituximab discontinuation at 5‐year analysis, Cox proportional hazards regression was performed using forward selection and backward elimination, with *P* < 0.20 associated with the deviance used for inclusion in and exclusion from the model. The proportional hazard assumption was tested by examining the Schoenfeld residuals plots. All statistical analysis was performed using Stata MP version 16 and Graph Pad Prism version 8 for Windows.

## RESULTS

### Patient and treatment characteristics

**Figure 1:**
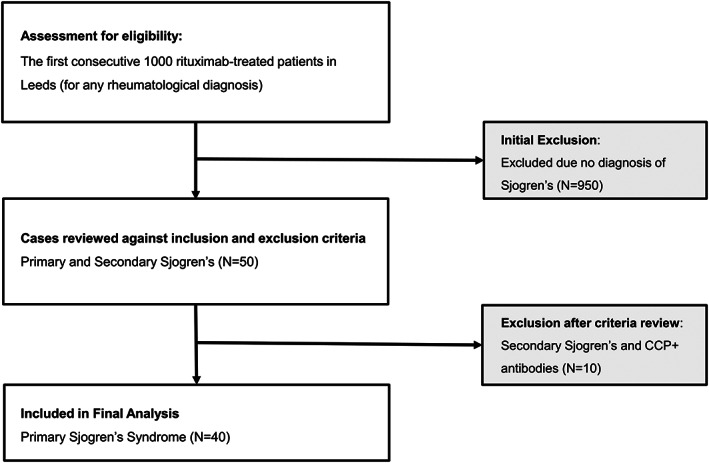
Flow chart of 40 patients with primary Sjögren syndrome included in the study. CCP, cyclic citrullinated peptide.

The flow of participant is illustrated in Figure 1. Of the 1000 rituximab‐treated patients, 40 had a diagnosis of pSS without RA overlap and were included in the analysis.

The baseline clinical characteristics and laboratory measures are described in Table 1. Twenty‐eight of 40 (70%) patients were on concomitant immunosuppressant (azathioprine, methotrexate, or mycophenolate mofetil [MMF]), the mean (SD) ESSDAI at rituximab initiation was 11.5 (6.7), 38/40 had at least moderate disease activity (ie, ESSDAI score ≥6), and the main indications for rituximab therapy were articular (73%), biological (50%), mucocutaneous (23%), hematological (20%), and nervous system (18%).

**Table 1:** Baseline characteristics of 40 rituximab‐treated patients with extra‐glandular pSS

Clinical characteristics or laboratory measures	Values
Age at cycle 1 rituximab infusion, mean (SD), y	54 (13.7)
Female, N (%)	38 (95)
Ethnicity, N (%)	
White	33 (82.5)
South Asian	5 (12.5)
South East Asian	1 (2.5)
Afro Caribbean	1 (2.5)
Disease duration at rituximab infusion, median (IQR), y	5.3 (2–9)
Positive ANA at diagnosis, N (%)	39 (98)
Positive ENA antibodies at rituximab infusion, N (%)	
Anti‐Ro (60)	33 (83)
Anti‐La	29 (73)
Positive rheumatoid factor at rituximab infusion, N (%)	20 (50)
Low complement level (C3 and/or C4) at rituximab infusion, N (%)	7 (18)
Prior therapy with cyclophosphamide, N (%)	11 (28)
No. of prior immunosuppressant failure (including cyclophosphamide and plasma exchange but excluding steroid), median (range)	2 (0–5)
Concomitant immunosuppressant started within 3 mo of cycle 1 rituximab infusion, N (%)	28 (70)
Methotrexate	22 (55)
Mycophenolate mofetil	4 (10)
Azathioprine	2 (5)
Concomitant oral prednisolone, N (%)	24 (60)
Oral prednisolone dose at cycle 1 rituximab infusion, mean (SD), mg/d	8 (11)
ESSDAI domains with indication for RTX, N (%)	
Articular	29 (73)
Biological	20 (50)
Mucocutaneous	9 (23)
Hematological	8 (20)
Peripheral and central nervous system	7 (18)
Lungs	4 (10)
Muscular	3 (8)
Constitutional	3 (8)
Glands	3 (8)
Renal	1 (3)
Lymphadenopathy	1 (3)
ESSDAI at rituximab infusion, mean (SD)	11.5 (6.7)
ESSDAI score <6 points, N (%)	2 (5)
ESSDAI score 6‐13 points, N (%)	29 (72.5)
ESSDAI ≥14 points, N (%)	9 (22.5)
CRP at rituximab infusion, median (IQR), mg/L	15 (6–45)
Immunoglobulin level at rituximab infusion, mean (SD), g/dL	
IgM (normal range 0.5‐2.0 g/L)	1.84 (1.62)
IgA (normal range 0.8‐4.0 g/L)	3.29 (1.82)
IgG (normal range 6.0‐16.0 g/L)	16.45 (6.41)
Peripheral blood B‐cell subsets, median (IQR), counts × 10^9^/L	
Naïve B cell	0.0950 (0.0313–0.1210)
Memory B cell	0.0130 (0.0056–0.0230)
Plasmablast cell	0.0026 (0.0010‐0.0055)

Abbreviations: ANA, antinuclear antibody; CRP, C‐Reactive Protein; ENA, extract nuclear antigen; ESSDAI, EULAR Sjögren syndrome disease activity; IQR, interquartile range; pSS, primary Sjögren syndrome; RTX, rituximab.

One hundred sixty‐nine rituximab cycles were administered with a total follow‐up of 165 patient‐years (PYs). The median (IQR) time to retreatment in rituximab responders for cycles 1 to 5 (C1‐C5) were 40 (31‐60), 43 (34–47), 40 (35‐59), 44 (38‐72), and 42 (35–53) weeks, respectively. Median (IQR) follow‐up per patient was 3 (1–6) years.

### Cycle 1: clinical and immunological response

**Figure 2:**
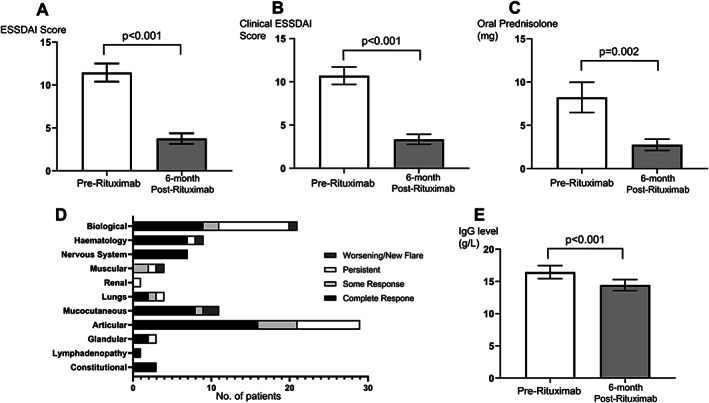
Clinical and immunological responses to cycle 1 rituximab therapy in pSS. Clinical measures before and 6 months after rituximab were assessed using **(A)** the ESSDAI, **(B)** clinical ESSDAI (without biological domains), and **(C)** oral prednisolone dose. **(D)** The number of patients with various degree of responses or a new flare at 6 months in the 11 main ESSDAI domains. **(E)** IgG levels (g/L) were compared before and after rituximab. Bar chart in A‐C and E denote mean and error bars show standard error of the mean. Paired student *t* test were used for comparison. CRP, C‐Reactive Protein; ESSDAI, EULAR Sjögren syndrome disease activity index; pSS, primary Sjögren syndrome; RTX, rituximab.

In C1 rituximab, there was a high degree of clinical response; the proportion of patient achieving ESSDAI response from baseline was 29/40 (73%; 95% confidence interval [CI]: 58‐87). There were significant reductions in ESSDAI score (mean difference −7.8 [95% CI −9.9 to −5.5]; *P* < 0.001; Figure 2A), clinical ESSDAI score (mean difference −7.35 [−9.52 to −5.81]; *P* < 0.001 (Figure 2B), and daily oral prednisolone dose (mean difference −5.5 [−8.8 to −2.2] mg; *P* = 0.002; Figure 2C) at 6 months from rituximab baseline. Of the 24/40 patients who were on concomitant oral prednisolone, 10/24 (42%) stopped steroid therapy at 6 months. The domains with at least 50% complete response rates post‐rituximab were hematological, nervous system, lungs, mucocutaneous, articular, glandular, lymphadenopathy, and constitutional. Worsening or new flare at 6 months occurred in the biological, hematological, muscular, and mucocutaneous domains (Figure 2D).

In terms of immunological response, there was a significant reduction in IgG levels at 6 months from baseline (mean difference −2.01 [−2.72 to −1.30] g/L; *P* < 0.001; Figure 2E). Paired pre‐ and post‐rituximab complement levels were available in 33/40 patients. Of the 6/33 patients with low complement at baseline, 3/6 (50%) had their levels normalized at 6 months.

### B‐cell kinetics across autoimmune diseases

**Figure 3:**
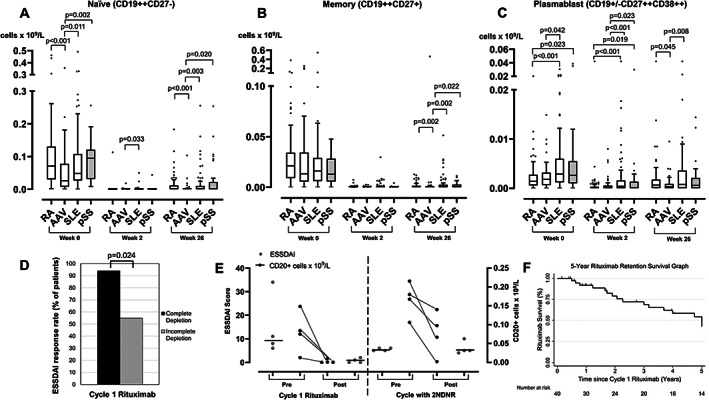
Comparison of peripheral B‐cell subsets across four diseases and associations with response. B‐cell subsets including naïve **(A)**, memory **(B)**, and plasmablast **(C)** were compared between patients with RA, AAV, SLE, and pSS at rituximab initiation, 2 weeks and 26 weeks post‐rituximab. The box plots denote median, and the error bars represent Tukeys. Analyses were performed using Kruskal‐Wallis followed by multiple non‐parametric testing with Dunn's correction. **(D)** The bar chart represents the proportion of patients with ESSSDAI response based on complete B‐cell depletion status. **(E)** The ESSDAI score (left Y‐axis and in grey dots) and CD20+ B cells (right Y‐axis and in black dots) are plotted for all four patients with 2NDNR to rituximab. The black horizontal line in the ESDDAI figure represents the median. **(F)** Kaplan‐Meier survival graph of rituximab retention at 5 years. The vertical markings on the graph denote censored cases. 2NDNR, secondary non‐depletion and non‐response; AAV, anti‐neutrophil cytoplasmic antibody‐associated vasculitis; ESSDAI, EULAR Sjögren syndrome disease activity; pSS, primary Sjögren syndrome; RA, rheumatoid arthritis; SLE, systemic lupus erythematosus.

We next compared longitudinal B‐cell subsets during rituximab therapy for pSS with the treatment of RA, SLE, and AAV. At rituximab baseline, naïve lymphopenia was shown to be a B‐cell–specific marker for active disease in AAV compared with RA, SLE, and pSS (Kruskal‐Wallis test, *P* < 0.001). At 2 weeks post‐rituximab, naïve B cells differed between groups (Kruskal‐Wallis test, *P* = 0.032), and this difference was due to higher counts in SLE versus AAV (*P* = 0.033). At 26 weeks post‐rituximab, patients with AAV had the lowest naïve B‐cell counts compared with patients with RA, SLE, and pSS (Kruskal‐Wallis test, *P* < 0.001) (Figure 3A).

For memory B cell, there was no difference between groups both at rituximab baseline and 2 weeks post‐rituximab. At 26 weeks post‐rituximab, memory B cells differed between groups (Kruskal‐Wallis test, *P* = 0.005); patients with AAV had the lowest counts compared with patients with RA, SLE, and pSS (Figure 3B).

Higher plasmablasts appeared to be a B‐cell–specific marker for active disease in SLE and pSS compared with RA and AAV (Kruskal‐Wallis test, *P* < 0.001). At 2 weeks post‐rituximab, plasmablast depletion were the least efficient in SLE and pSS compared with RA and AAV (Kruskal‐Wallis test, *P* < 0.001). At 26 weeks post‐rituximab, plasmablasts differed between groups (Kruskal‐Wallis test, *P* = 0.010); patients with AAV had the lowest counts compared with patients with RA and SLE (Figure 3C). In summary, the longitudinal B‐cell subset profile in pSS was most similar to SLE and distinct from both RA and AAV.

### Association between complete B‐cell depletion and clinical response

Of 39/40 patients with complete data, 17/39 (43.6%) achieved complete depletion at 2 weeks post‐rituximab. Complete B‐cell depletion was associated with ESSDAI response at 6 months (complete = 16/17 [94.1%] vs. incomplete depletion = 13/22 [59.1%]; *P* = 0.024) (Figure 3D). There was no association between concomitant azathioprine/MMF versus methotrexate/None and achieving complete B‐cell depletion (50% versus 42% respectively; *P* = 1.00).

### Retreatment of first cycle non‐responders

In RA, we showed that retreatment of initial non‐responders with incomplete B‐cell depletion led to improved response rate (72%) in C2 (32). In contrast to our findings in RA, in pSS, 9/11 non‐responders were retreated, but only 2/9 responded in C2 regardless of whether complete depletion was achieved in the subsequent cycle.

### Retreatment of first cycle responders

**Figure 4:**
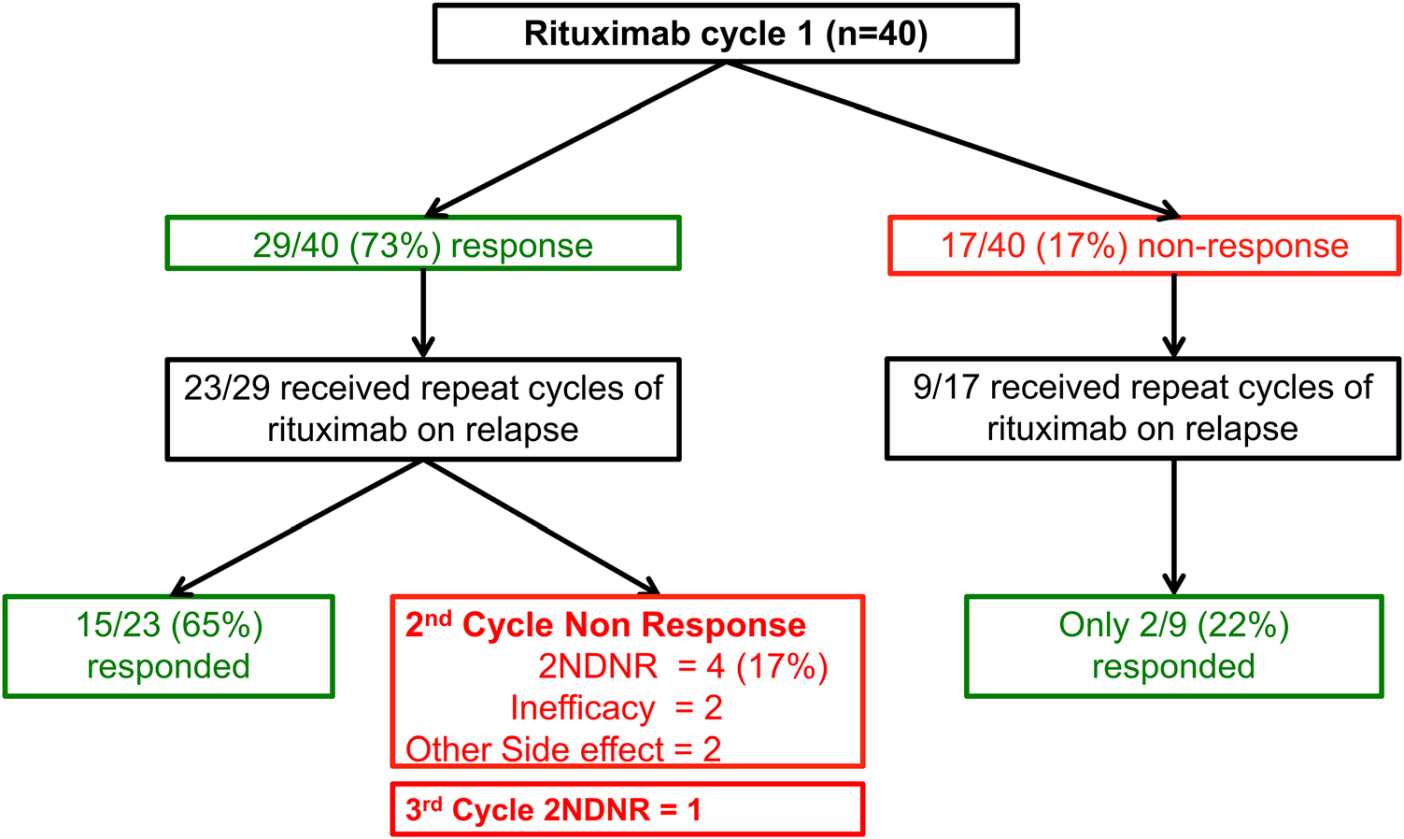
Flow chart of effectiveness of repeat cycles with rituximab in extra‐glandular primary Sjögren syndrome. 2NDNR, secondary non‐depletion and non‐response.

Of C1 responders, 23/29 received retreatment on clinical relapse. Of these, 15/23 (65%) patients responded, whereas 8/23 (35%) lost response in C2 (2NDNR = 4/23 [17.3%]; inefficacy = 2; other side effects = 2; Figure 4). Data for B cells and ESSDAI scores for the four patients who met the 2NDNR criteria are illustrated in Figure 3E.

### Factors predicting sustained clinical response (first two cycles)

Within the two rituximab cycles, 27/40 (68%) patients continued therapy. We defined sustained response as ESSDAI response in both cycles. Patients who discontinued therapy after one cycle due to non‐response or toxicity were classified as non‐sustained responders. In multivariable analysis, concomitant immunosuppressant (odds ratio 7.16 [95% CI: 1.37‐37.35]; *P* = 0.019) and achieving compete B‐cell depletion in C1 (9.78 [95% CI: 1.32‐72.25]; *P* = 0.025) were associated with increased odds of ESSDAI response in both rituximab cycles (Table 2).

**Table 2:** Multivariable analysis with multiple imputation of predictors of short‐term response to rituximab in pSS

Predictors	Continued response (N = 27)	Non‐response within 2 RTX cycles (N = 13)	Univariable OR (95% CI); *P* value	Multivariable OR (95% CI); *P* value
Age, mean (SD), per 10 y	54.8 (14)	52.2 (12.5)	1.15 (0.71–1.87); 0.572	Not included in MVA
Non‐Caucasian vs. Caucasian (Ref), %	22.2%	7.7%	3.43 (0.37–31.97); 0.279	Not included in MVA
Disease duration, median (IQR), y	5.3 (1.6–8.7)	6.2 (3.3–9.2)	1.03 (0.93–1.15); 0.565	Not included in MVA
Concomitant immunosuppressant, %	81.5%	30.8%	**9.90 (2.15–45.56); 0.003**	**7.16 (1.37–37.35); 0.019**
Concomitant oral prednisolone^a^, %	66.7%	46.2%	2.33 (0.61–9.02); 0.220	Not included in MVA
IgG, mean (SD), g/L	15.5 (6.3)	18.5 (5.8)	0.93 (0.83–1.03); 0.175	Included in MVA but excluded in final model since *P* > 0.20
Clinical ESSDAI score, mean (SD)	11.2 (5.7)	9.7 (7.2)	1.04 (0.93–1.17); 0.487	Not included in MVA
High activity (ESSDAI ≥14) vs. ESSDAI <14 score (Ref), %	29.6%	7.7%	5.05 (0.56–45.64); 0.149	Included in MVA but excluded in final model since *P* > 0.20
Baseline naïve B cells, median (IQR), counts × 10^9^/L^b^	96 (35–110)	110 (62–121)	1.00 (0.99–1.01); 0.856	Not included in MVA
Baseline memory B cells, median (IQR), counts × 10^9^/L^b^	13 (7–27)	13 (5–29)	1.00 (0.95–1.05); 0.976	Not included in MVA
Baseline plasmablasts, median (IQR), counts × 10^9^/L^b^	3 (1–5)	2.3 (1.6–9.5)	0.99 (0.93–1.06); 0.779	Not included in MVA
Complete B‐cell depletion in previous rituximab cycle, %	59.3%	7.7%	**17.45 (1.97–154.36); 0.010**	**9.78 (1.32–72.25); 0.025**

*Note*: The Bolding indicate variables with significant association with short‐term response to rituximab.

Abbreviations: CI, confidence interval; ESSDAI, EULAR Sjögren syndrome disease activity; IQR, interquartile range; MMF, mycophenolate mofetil; MVA, multivariable analysis; OR, odds ratio; pSS, primary Sjögren syndrome; RTX, rituximab.

^a^ Concomitant immunosuppressant was defined as on either azathioprine, methotrexate, or mycophenolate mofetil. In univariable analyses, the effect of concomitant methotrexate on short‐term response was OR 7.2 (95% CI: 1.53–33.85; *P* = 0.012), whereas all six patients who were on azathioprine/MMF responded to the first two rituximab cycles (predicted fully). Hence, due to no statistically significant difference in immunosuppressant type and our sample size, concomitant immunosuppressant as a whole was evaluated in the multivariable analysis.

^b^ Counts multiplied by 1000 prior to multivariable analysis due to extreme decimal points of the original values.

### Longer‐term effectiveness of rituximab

After 5 years, 17/40 (43%) patients had discontinued rituximab. The causes for discontinuation were inefficacy (7), 2NDNR (5), other side effects (2), and deaths (3) (pneumonia = 1; hypoxic brain injury from an out of hospital cardiac arrest = 1; frailty and pressure sores = 1). One additional patient had 2NDNR in C3 of rituximab. The 5‐year rituximab retention Kaplan‐Meier survival graph is illustrated in Figure 3F.

### Factors associated with 5‐year rituximab retention

Predictors of time to rituximab discontinuation at 5 years was analyzed. In univariable analyses, concomitant immunosuppressant (hazard ratio 0.17 [95% CI: 0.06–0.47]; *P* = 0.001) and achieving compete B‐cell depletion in C1 (0.24 [95% CI: 0.07–0.83]; *P* = 0.024) were associated with reduced risk of therapy discontinuation. In addition to the two variables above, we also included baseline plasmablasts (*P* < 0.20 in univariable analysis) and high activity (ESSDAI ≥14) versus ESSDAI score of less than 14 score (ie, an important clinical factor) in the multivariable modelling. In the multivariable analysis, only concomitant immunosuppressant (0.14 [0.05–0.46]; *P* = 0.001) was associated with reduced risk (Supplementary Table 1).

### Long‐term safety of rituximab in pSS

There were 12 severe infection episodes (SIEs) in 10 patients (7.3/100 PYs; pneumonia = 6; cellulitis and osteomyelitis = 4; COVID‐19 pneumonitis with subsequent recovery = 2). These breakthrough COVID‐19 pneumonitis (both patients were double‐vaccinated) occurred at 2 and 9 months after C5 and C1 of rituximab, respectively. Both had multiple comorbidities, including interstitial lung disease, pulmonary hypertension, and a previous history of tuberculosis in the thyroid, and were on concomitant oral prednisolone. At the end of the follow‐up, 2/40 (5%) had secondary hypogammaglobulinemia of which one had a low IgG level preceding rituximab therapy. Both patients subsequently suffered from recurrent infections and required treatment with immunoglobulin replacement (occurring from cycles 3 and 14, respectively). There was no case of lymphoma observed post‐rituximab, but 1/40 (3%) patient developed CD5‐negative lymphoproliferative disorder at 5 years since her last rituximab infusion.

## DISCUSSION

The clinical challenges for the use of B‐cell–depleting therapy with rituximab in pSS include defining subgroups of patients likely to respond to the initial and subsequent cycles of therapy. By capturing data of a broad spectrum of patients with pSS treated with rituximab with matched B‐cell data for biological response, as well as long‐term follow‐up, this study offers insights into pragmatic use of rituximab and has implications for the future development of anti‐CD20 antibodies.

In this study, we reported a high degree of clinical response (73%) to rituximab in the initial cycle. Our cohort comprised patients with moderate to severe disease activity. Additionally, over a quarter had either relapsed or were refractory to cyclophosphamide therapy. The domains most likely to respond included hematological, lungs, articular, glandular, lymphadenopathy, and biological, which concurred with findings from other studies (16, 33). In the French Autoimmunity and Rituximab (AIR) registry, the majority of patients with vasculitis or sensory‐motor peripheral neuropathy responded to rituximab, whereas the patients with pure sensory neuropathy did not respond (16). In contrast, all our 7/40 patients with neurological manifestations (with or without vasculitis) responded to rituximab (sensory neuronopathy/ganglionopathy = 2; sensory‐motor polyneuropathy = 2; cryoglobulinemic neuropathy = 1; cerebral vasculitis = 1; transverse myelitis = 1). We also observed good response in the constitutional and mucocutaneous domains (including three patients with ulcers related to vasculitis), although in the latter, two patients had a new subacute cutaneous lupus flare post‐rituximab. This temporary cutaneous flare has also been observed in SLE, which could indicate a change in immune regulation following B‐cell–depleting therapy (34). At 5 years, the maintenance of rituximab was just over 50%, which is a good outcome from a B‐cell–depleting therapeutics perspective.

In terms of biological response, in addition to the routine immunology tests, we measured peripheral blood B cells pre‐ and post‐therapy using HSFC. Only 43.6% of patients achieved complete B‐cell depletion at 2 weeks post‐rituximab. This is the first study to show that achieving complete depletion after one rituximab infusion was associated with a superior outcome in pSS and concurred with our findings and others in RA (18, 35). Analyses of B‐cell subsets across four B‐cell–mediated autoimmune diseases provided a more nuanced disease‐specific signature. We showed that higher plasmablasts is a marker of active disease for pSS and SLE. Depletion of plasmablast cells at 2 weeks was also often incomplete in both diseases compared with RA and AAV. Hence the longitudinal B‐cell subset profile of pSS resembles that of SLE, and not RA and AAV. Therefore, as in SLE, future therapeutics should aim to achieve complete B‐cell depletion. Treatment modification could be employed to improve depletion either by increasing the dose, adding an extra infusion in patients with incomplete B‐cell depletion post‐rituximab as our group previously showed in RA (26), or the use of type 2 anti‐CD20 antibodies with enhanced antibody‐dependent cellular cytotoxicity, such as obinutuzumab as in SLE. Genotyping for the Fc γ receptor IIIA polymorphism on the position 158 (*FCGR3A*‐158V), which encodes for high affinity for IgG1 antibodies, as well as quantitative analysis of copy number variant, may help stratify patients for optimal dose and treatment protocols as shown in both RA and SLE (36, 37, 38).

In repeat cycles of rituximab, the incidence of 2NDNR in our pSS cohort (17.4%) was similar to SLE (12%) (20). The French AIR registry also reported that 5/41 (12.2%) patients with pSS had severe infusion reactions or serum sickness in repeat cycles, which led to rituximab discontinuation. This could be in keeping with the 2NDNR phenomenon although B cells were not measured in their study (16). Another study also reported that antidrug antibody against rituximab was more frequent in systemic autoimmune diseases (14.7%; consisted of 38/75 patients with pSS) than in RA (2.4%). Patients developing antidrug antibody had frequently infusion reactions at the second infusion or during further cycles, which is in keeping with our 2NDNR definition. This study also identified African ancestry as a predictor of immunogenicity to rituximab (39). Our pSS cohort only consisted of one patient of African ancestry, and this patient did not experience a 2NDNR. All five patients with 2NDNR were of European ancestry. Interestingly, in our case series of a United Kingdom multicenter cohort in SLE, 2/9 and 5/9 patients with 2NDNR to rituximab and subsequently treated with obinutuzumab were of African and South Asian ancestry, respectively (40). Thus, inclusion of a larger number of patients of non‐European ancestry may explore the effect of ancestry on the development of antidrug antibody to rituximab. For patients with 2NDNR, we and others have previously showed that switching to a humanized or type 2 anti‐CD20 antibodies restored depletion and response in SLE and AAV (20, 21, 22, 24). In this study, we also identified two predictors of sustained response (within two rituximab cycles) and rituximab retention at 5 years, which were the concomitant use of immunosuppressant and achieving complete B‐cell depletion in the previous cycle.

No new major safety signals from rituximab therapy during our long‐term follow‐up. Our SIE rate (7.3/100 PYs) was higher compared with data from RCTs and long‐term extension studies of rituximab in RA (3.94/100 PYs) (41) and registries of rituximab in pSS (1.3/100 PYs), but comparable to registries in systemic autoimmune rheumatic diseases (range 5.0‐17.1/100 PYs) (42, 43, 44). The high rate of SIE in our cohort could be attributed to patients comprising those with multiple comorbidities, high disease burden, and that nearly two thirds were on concomitant glucocorticoids. Furthermore, efficacy RCTs (and meta‐analyses derived from them) are of limited value in capturing SIEs in those with multiple comorbidities (45). Higher prerituximab IgG level is a marker of active pSS and can be an advantage regarding long‐term safety of therapy. Nevertheless, repeat cycles of rituximab in this study led to 5% of patients with recurrent infections being associated with hypogammaglobulinemia, who subsequently required treatment with immunoglobulin replacement. Although the rate of immunoglobulin replacement requirement in our pSS cohort is lower compared with up to 11% in other diseases such as AAV (46), immunoglobulin (IgM, IgA, and IgG) levels need to be monitored before and after rituximab therapy to make an informed decision about SIE risk during repeat cycles (44, 47). Part of our study follow‐up was also during the height of the COVID‐19 pandemic, which is a specific safety issue for rituximab‐treated patients that was not present during previous reports. The breakthrough COVID‐19 infection cases despite vaccination in this cohort also highlight the need to be vigilant in scheduling rituximab therapy; all patients are currently recommended a third primary and a booster vaccination dose in view of a poor seroconversion rate in rituximab‐treated autoimmune rheumatic diseases, administering vaccination at least 6 months after rituximab administration, or the use of passive immunity instead (48, 49).

This study has some limitations. First, an interobserver variability could have occurred in the ESSDAI assessments because of the lengthy follow‐up duration and a cohort that was highly heterogeneous in pSS manifestations. However, all patients were managed in a dedicated single center, cases were adjudicated by a group of experts who were involved in the pivotal rituximab RCTs, and a comprehensive case notes and laboratory results review were performed in all patients to complement our adjudication. Second, because of its retrospective design, data pertaining to the patient‐reported outcome such as the EULAR Sjögren Patient Reported Index were not captured routinely in our practice but nevertheless, could be useful aid in the response prediction in future studies. Third, concomitant therapy with immunosuppressant was used in 70% of the patients, thus effectiveness could not be attributed solely to rituximab, although patients only received rituximab because these immunosuppressants had been ineffective as monotherapy. Fourth, the lack of a control group limits interpretation of effectiveness and safety of rituximab. We used a decrease in ESSDAI score of 3 or more as our endpoint as this has been defined as minimal clinically important improvement in previous literature (50). We are aware that in recent RCTs in pSS, the placebo responses as defined by a decrease in ESSDAI score ranges from approximately 3 to 7 (51, 52). Importantly, none of our ESSDAI responders had to switch to therapy because of treatment failure. Finally, we did not specifically measure the antirituximab antibody in patients with 2NDNR. In our previous study in SLE, we showed that all patients with 2NDNR had detectable antirituximab antibody. However, in those who continued to respond to the repeat rituximab cycles, 56% also had detectable antirituximab antibody (20). Therefore, measuring the antirituximab antibody alone is not enough to identify patients as 2NDNR. Instead, clinical features, ie, severe infusion reaction and non‐response and measuring B cells, are more meaningful. Our previous findings above may also explain why measuring antirituximab antibody is not routinely performed in clinical practice in pSS and SLE compared with other antichimeric antibody such as infliximab, where antiinfliximab antibody and infliximab trough level are more specific in predicting imminent loss of response to infliximab in inflammatory bowel disease (53), RA, and axial spondyloarthropathy (54, 55).

In conclusion, treatment for systemic manifestations of pSS with rituximab is highly effective and can be guided by B‐cell monitoring with the aim of achieving complete depletion. About one in six patients with pSS lose depletion on repeat cycles of rituximab due to 2NDNR, which has previously been associated with antirituximab antibodies. Concomitant use of oral immunosuppressant and alternative humanized and/or type 2 anti‐CD20 antibodies may improve outcomes of first and repeat cycles of B‐cell depletion in pSS; definitive studies are therefore warranted in extra‐glandular pSS.

## AUTHOR CONTRIBUTIONS

All authors were involved in drafting the article or revising it critically for important intellectual content, and all authors approved the final version to be published. All authors have agreed to be accountable for all aspects of the work, had full access to all of the data in the study, and take responsibility for the integrity of the data and the accuracy of the data analysis.

### Study conception and design

Pepple, Arnold, Vital, Emery, Md Yusof.

### Acquisition of data

Pepple, Arnold, Vital, Emery, Md Yusof.

### Analysis and interpretation of data

Pepple, Arnold, Vital, Rawstron, Pease, Dass, Emery, Md Yusof.

## Supporting information


**Disclosure Form**:  Click here for additional data file.

**Table S1** Multivariable analysis with multiple imputation of predictors of time‐to‐rituximab discontinuation at 5 years in pSS
