# Supplementary material for: Predicting Sustained Clinical Response to Rituximab in Moderate to Severe Systemic Manifestations of Primary Sjögren Syndrome
Source: ACR Open Rheumatol. 2022 Jun 5;4(8):689–99. doi: 10.1002/acr2.11466 (PMC9374056; doi:10.1002/acr2.11466)
Supplement: Supplementary file 2 — Table S1 Multivariable analysis with multiple imputation of predictors of time‐to‐rituximab discontinuation at 5 years in pSS [file ACR2-4-689-s002.docx]

**SUPPLEMENTARY DATA**

**Table S1: Multivariable analysis with multiple imputation of predictors of time-to-rituximab discontinuation at 5 years in pSS**

| **Predictors** | **Univariable analysis**  **HR (95% CI); p-values (with multiple imputation)** | **Multivariable analysis**  **HR (95% CI); p-values (with multiple imputation)** |
| --- | --- | --- |
| Age, mean (SD), per 10 years | 0.83 (0.60-1.13); 0.235 | Not included in MVA |
| Non-Caucasian vs Caucasian (Ref), | 0.83 (0.19-3.64); 0.805 | Not included in MVA |
| Disease duration, per year | 0.96 (0.88-1.05); 0.397 | Not included in MVA |
| Concomitant immunosuppressant, | **0.17 (0.06-0.47); 0.001** | **0.14 (0.05-0.46); 0.001** |
| Concomitant oral prednisolone, | 0.88 (0.34-2.29); 0.798 | Not included in MVA |
| IgG, mean (SD), per g/L | 1.02 (0.95-1.09); 0.569 | Not included in MVA |
| Clinical ESSDAI score, per point | 0.97 (0.88-1.06); 0.473 | Not included in MVA |
| High activity (ESSDAI≥14) vs ESSDAI<14 score (Ref) | 0.39 (0.09-1.71); 0.213 | Included in MVA but excluded in final model since p>0.20 |
| Baseline Naïve B-cells, counts x 10^9^/L** | 1.00 (0.99-1.01); 0.215 | Not included in MVA |
| Baseline Memory B-cells, counts x 10^9^/L** | 1.01 (0.97-1.04); 0.767 | Not included in MVA |
| Baseline Plasmablasts, counts x 10^9^/L** | 1.05 (0.99-1.11); 0.112 | 1.06 (0.98-1.15); 0.136 |
| Complete B-cell depletion in previous rituximab cycle | **0.24 (0.07-0.83); 0.024** | 0.32 (0.09-1.16); 0.083 |
